# The phenotypic spectrum and genotype-phenotype correlations in 106 patients with variants in major autism gene *CHD8*

**DOI:** 10.1038/s41398-022-02189-1

**Published:** 2022-10-01

**Authors:** Alexander J. M. Dingemans, Kim M. G. Truijen, Sam van de Ven, Raphael Bernier, Ernie M. H. F. Bongers, Arjan Bouman, Laura de Graaff – Herder, Evan E. Eichler, Erica H. Gerkes, Christa M. De Geus, Johanna M. van Hagen, Philip R. Jansen, Jennifer Kerkhof, Anneke J. A. Kievit, Tjitske Kleefstra, Saskia M. Maas, Stella A. de Man, Haley McConkey, Wesley G. Patterson, Amy T. Dobson, Eloise J. Prijoles, Bekim Sadikovic, Raissa Relator, Roger E. Stevenson, Connie T. R. M. Stumpel, Malou Heijligers, Kyra E. Stuurman, Katharina Löhner, Shimriet Zeidler, Jennifer A. Lee, Amanda Lindy, Fanggeng Zou, Matthew L. Tedder, Lisenka E. L. M. Vissers, Bert B. A. de Vries

**Affiliations:** 1 Department of Human Genetics, Donders Institute for Brain, Cognition and Behaviour, Radboud University Medical Center, P.O. Box 9101, 6500 HB Nijmegen, the Netherlands; 2 Department of Psychiatry and Behavioral Sciences, University of Washington, Seattle, WA USA; 3 Department of Clinical Genetics, Erasmus MC, University Medical Center Rotterdam, Rotterdam, The Netherlands; 4 Department of Genome Sciences, University of Washington, Seattle, WA USA; 5 Howard Hughes Medical Institute, University of Washington, Seattle, WA USA; 6 Department of Genetics, University of Groningen, University Medical Center Groningen, Groningen, The Netherlands; 7 Department of Human Genetics, Amsterdam UMC location Vrije Universiteit Amsterdam, Boelelaan 1117, Amsterdam, The Netherlands; 8 Department of Complex Trait Genetics, Center for Neurogenomics and Cognitive Research, VU University, Amsterdam, the Netherlands; 9 1Verspeeten Clinical Genome Centre, London Health Sciences Centre, London, ON N6A 5W9 Canada; 10 Department of Clinical Genetics, Amsterdam UMC, University of Amsterdam, Amsterdam, the Netherlands; 11 Department of Pediatrics, Amphia Hospital, Breda, The Netherlands; 12 Greenwood Genetic Center, Greenwood, SC 29646 USA; 13 Department of Pathology and Laboratory Medicine, Western University, London, ON N6A3K7 Canada; 14 Department of Clinical Genetics, MUMC, Maastricht, The Netherlands; 15 GROW-School for Oncology and Reproduction, Maastricht University, Maastricht, Netherlands; 16 GeneDx, 207 Perry Parkway, Gaithersburg, MD 20877 USA

**Keywords:** Autism spectrum disorders, Clinical genetics

## Abstract

*CHD8*, a major autism gene, functions in chromatin remodelling and has various roles involving several biological pathways. Therefore, unsurprisingly, previous studies have shown that intellectual developmental disorder with autism and macrocephaly (IDDAM), the syndrome caused by pathogenic variants in *CHD8*, consists of a broad range of phenotypic abnormalities. We collected and reviewed 106 individuals with IDDAM, including 36 individuals not previously published, thus enabling thorough genotype–phenotype analyses, involving the *CHD8* mutation spectrum, characterization of the *CHD8* DNA methylation episignature, and the systematic analysis of phenotypes collected in Human Phenotype Ontology (HPO). We identified 29 unique nonsense, 25 frameshift, 24 missense, and 12 splice site variants. Furthermore, two unique inframe deletions, one larger deletion (exons 26–28), and one translocation were observed. Methylation analysis was performed for 13 patients, 11 of which showed the previously established episignature for IDDAM (85%) associated with *CHD8* haploinsufficiency, one analysis was inconclusive, and one showing a possible gain-of-function signature instead of the expected haploinsufficiency signature was observed. Consistent with previous studies, phenotypical abnormalities affected multiple organ systems. Many neurological abnormalities, like intellectual disability (68%) and hypotonia (29%) were observed, as well as a wide variety of behavioural abnormalities (88%). Most frequently observed behavioural problems included autism spectrum disorder (76%), short attention span (32%), abnormal social behaviour (31%), sleep disturbance (29%) and impaired social interactions (28%). Furthermore, abnormalities in the digestive (53%), musculoskeletal (79%) and genitourinary systems (18%) were noted. Although no significant difference in severity was observed between males and females, individuals with a missense variant were less severely affected. Our study provides an extensive review of all phenotypic abnormalities in patients with IDDAM and provides clinical recommendations, which will be of significant value to individuals with a pathogenic variant in *CHD8*, their families, and clinicians as it gives a more refined insight into the clinical and molecular spectrum of IDDAM, which is essential for accurate care and counselling.

## Introduction

The chromatin helicase DNA-binding (CHD) gene family functions in the ATP-dependent chromatin remodelling of proteins, which plays a vital role in regulating gene transcription [1]. One of the family members, chromodomain helicase DNA binding protein 8 (*CHD8*), is located at 14q11.2 and has been ascribed various roles involving several biological pathways, including a function in chromatin remodelling by binding to beta-catenin [1], regulating Wnt signalling [2], and regulating P53-mediated apoptosis by histone H1 recruitment during cell proliferation in early embryogenesis [3]. Animal research has confirmed the gene’s wide range of functions, with reported symptoms in *CHD8*-deficient zebrafish and mice, ranging from constipation to disrupted dorsal neuronal development and autism spectrum disorder (ASD)-like behavioural characteristics [4, 5].

A similar wide spectrum of clinical characteristics is observed in individuals with pathogenic variants in *CHD8* (named intellectual developmental disorder with autism and macrocephaly, or IDDAM, OMIM #610528), including ASD, macrocephaly, hypotonia, gastrointestinal problems, and early and rapid postnatal growth [4, 6, 7]. Furthermore, typical dysmorphic features in individuals with IDDAM include an increased occipitofrontal circumference (OFC), pronounced supraorbital ridges, wide-set eyes with down slanted palpebral fissures, a broad nose with full nasal tip, and a pointed chin [4]. Neurodevelopmental and behavioural problems have been reported as well, such as mild to severe intellectual disability (ID), delay in at least one of the primary developmental domains, a delay in the development of social skills, sleeping problems, and a range of stereotypic or repetitive behaviours as a part of ASD. Maintaining attention is a known problem, and attention-deficit/hyperactivity disorder (ADHD) has been reported in a few cases [8]. Neurological symptoms include seizures and coordination problems [6]. Interestingly, while there are sample data available on the clinical features [9], a thorough review of possible genotype-phenotype correlations in *CHD8* is lacking so far.

To investigate possible genotype–phenotype correlations, detailed phenotypic information, preferably systematically captured in Human Phenotype Ontology (HPO), is aligned with genetic data, including both variant information as well as a functional read-out. For the latter, DNA methylation signatures have gained attention in recent years. For several genetic syndromes, including IDDAM, specific DNA methylation “episignatures” or “EpiSigns” have been generated [10, 11], which can be used to support the pathogenicity of a genetic variant. Such functional read-out is of particular interest to classify variants of unknown significance (VUSs) [10].

Overall, this study aims to perform a detailed genotype-phenotype correlation study for CHD8, by reviewing the phenotypes and genotypes of 106 individuals with IDDAM, of whom 36 have not been reported before in the literature. In addition, for a subset of 13 individuals, an episignature analysis was performed to enable better classification of the observed genotype. The results will be essential in defining the phenotypic spectrum of IDDAM, caused by pathogenic variants in *CHD8*, which will be helpful to inform and guide individuals with a variant in this gene and their families.

## Materials and methods

### Data collection and extraction

To collect individuals with (likely) pathogenic *CHD8* variants/IDDAM, a broad literature search was performed using Pubmed, Embase, and Web of Science by two independent researchers (SV and AJMD) in June 2020. Articles concerning *CHD8* were screened based on title, abstract and full text. Clinical data were extracted from the original papers. Furthermore, clinicians were contacted to gather information on individuals with a variant in *CHD8* whose phenotype was not yet published. The clinicians were asked to fill in an extensive table with clinical details (Supplementary Table 1).

Individuals included in the literature study have an established pathogenic or likely pathogenic variant (American College of Medical Genetics and Genomics (ACMG) class 4 or 5 [12]) in *CHD8*. In order to clearly describe CHD8-specific genotype–phenotype relationships, individuals were excluded if another (possible) pathogenic variant (ACMG class 4 or 5) was found in another gene (dual diagnosis). For copy number variants (CNVs), the genes residing in the CNV were analysed to establish that only *CHD8* was involved.

Patient information was collected using the format of the Human Disease Genes (HDG) website series, an international library of websites for professional information about genes and clinical knowledge [13]. To collect the phenotypic data systematically, terms of the HPO [14] were used. St. Jude’s ProteinPaint was used to visualize the genetic variants identified in *CHD8* [15].

### Data analysis: comparing groups and disease severity

**Fig. 1:**
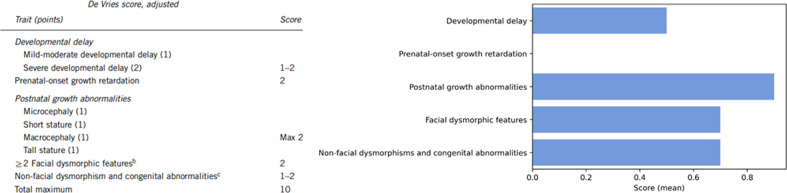
Adjusted De Vries score [16], with on the right the average scores of the different categories of the De Vries score. The scores are quite evenly distributed among the different categories—apart from prenatal-onset growth retardation, which was not present in our study cohort.

A possible genotype/phenotype correlation was investigated to determine a difference in phenotypic presentation between individuals with a missense variant and individuals with other variants (including nonsense, frameshift, splice-site variants and individuals with an inframe deletion, and the individuals with a translocation and an intragenic deletion). Furthermore, a possible difference in phenotype between males and females was investigated. To quantify disease severity, the adjusted De Vries score [16, 17] was used. The De Vries score was developed as a relatively simple phenotypic severity score for individuals with intellectual disability in which points are given for (severity of) intellectual disability, growth abnormalities (prenatal and postnatal), facial dysmorphisms, non-facial dysmorphisms, and other congenital anomalies (Fig. 1) [16]. A higher score indicates a more severe phenotype. Statistical significance of a difference in the De Vries score between groups was determined using the Mann–Whitney-*U* test, and a *p*-value lower than 0.05 was considered statistically significant. In this analysis, only individuals with a (likely) pathogenic variant were included.

### EpiSign analysis

DNA methylation analysis was performed using Illumina Infinium EPIC arrays. The methylation profile of each new sample was compared to 57 established episignatures associated with 65 conditions (in EpiSign v3) including IDDAM. The EpiSign Knowledge Database (EKD) is utilized in the analysis by comparing methylation profile of new clinical test samples to specific cohorts and reference controls in the EKD. Methylation pattern similarities between new samples and EKD samples were assessed using a support vector machine (SVM)-based classifier, where outputs, referred to as methylation variant pathogenicity (MVP) scores are values ranging from 0 to 1. The MVP scores represent the probability of predicting a test sample as positive for a specific condition, where higher scores indicate positive with high confidence. Classification results are further assessed and supported by unsupervised clustering using heatmap and multidimensional scaling (MDS) plots showing the test sample’s methylation data relative to the cohort cases and controls using the episignature probes. More detailed information about this standard analysis pipeline is discussed in previous works [11, 18, 19].

### Consent and ethical approval

Informed consent for participation in this study was obtained from the individuals or their legal guardians, respectively. The study was approved by the institutional review board of the Radboud University Medical Center (#2020-6764).

## Results

### Cohort

**Fig. 2:**
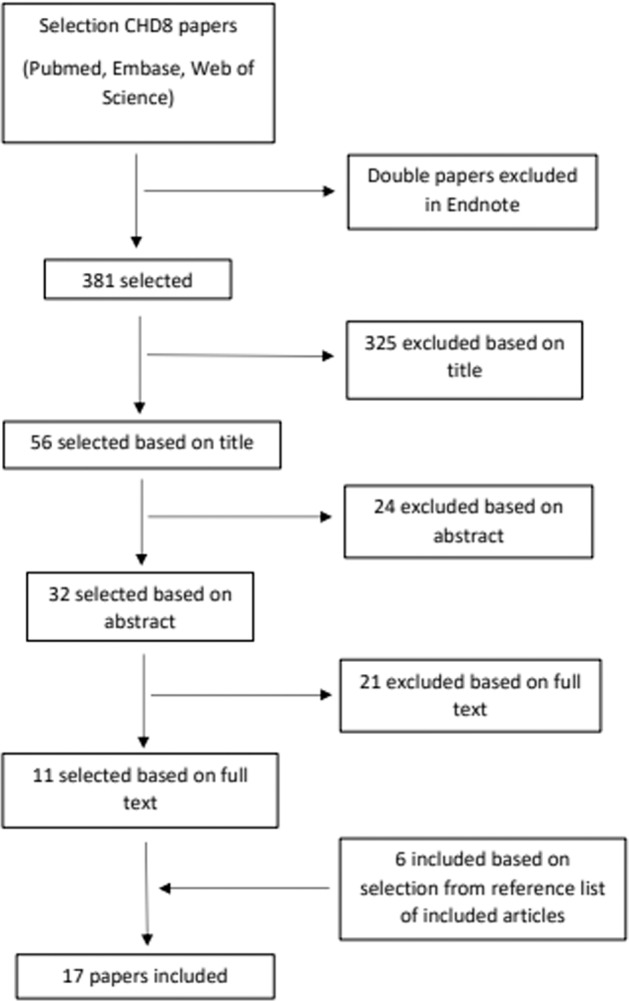
This PRISMA flowchart displays the workflow and selection of studies during the systematic review that was performed. Eleven studies were found using our search strategy and by looking at all references from those articles, another six could be included, leading to 17 papers included in our study.

The performed literature search yielded 381 articles, of which 17 articles were included (Fig. 2). These articles described the pheno- and genotypes of 70 individuals with a (likely) pathogenic variant in *CHD8*. Through international collaboration, data on 36 novel individuals could be added, of whom five individuals had a VUS, and 31 a (likely) pathogenic variant in *CHD8*. Our total cohort thus consisted of a total of 106 individuals; 76 of the individuals in the cohort were male and 30 were female. The median age was 7 years (range: 1–57 years).

### Genotype

**Fig. 3:**
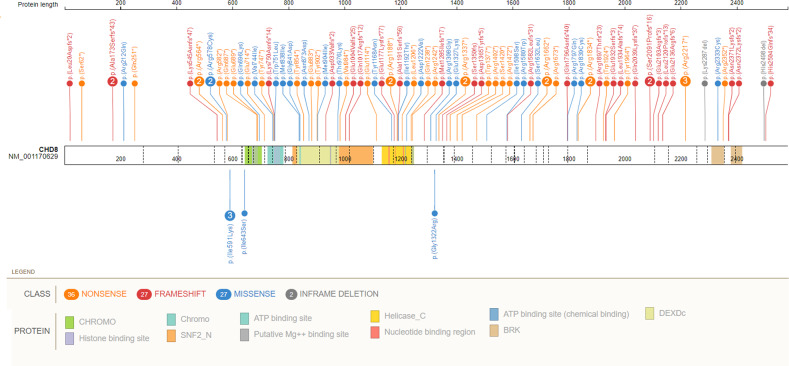
Variants found in *CHD8*. The splice site variants, exon deletion and translocation are not shown. Above: all (likely) pathogenic nonsense-, frameshift- and missense variants, with the two inframe deletions added as well. The missense variants are rather spread out over the gene and there does not seem to be a clear relation with a specific domain or protein function, as sometimes is the case (and could help with assessing pathogenicity). Below are the five variants of which pathogenicity is unknown, including the two with a different methylation signature. Dashed lines indicate the different exons.

In 106 individuals, 29 unique nonsense, 25 frameshift, 24 missense, and 12 splice-site variants were observed (Fig. 3, Supplemental Table 1). Furthermore, two unique in-frame deletions, one larger exon deletion and one translocation (in which *CHD8* was the only gene disrupted) were observed. Variants were spread across the entire gene, although a concentration of variants was seemingly present in the N-terminal part of the protein.

### EpiSign analysis

**Fig. 4:**
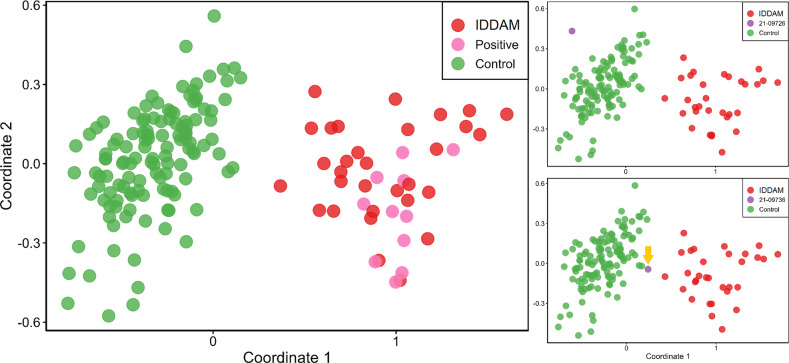
The multidimensional scaling (MDS) plots of the methylation data are shown here. In the left diagram, 11 positive test cases (pink) are displayed when plotted against affected individuals with IDDAM and a control group. These 11 cases clearly cluster within the IDDAM group and not in the control group. The other two individuals investigated with methylation analysis are displayed in the right images, with one (top-right) test sample with a possible gain of function (purple), and (bottom-right) an inconclusive test sample plotted with IDDAM signature cases (red) and reference controls (green).

EpiSign analysis was performed on DNA samples available from 13 patients. MVP scores using the IDDAM signature SVM model and clustering plots with reference IDDAM signature samples and probes were assessed for each test data (Fig. 4). All 13 test samples were negative for the 56/57 non-IDDAM signature. Eleven of the 13 individuals (85%) were classified as positive for IDDAM with high confidence. One individual (with variant c.1928T>G, p.(Ile643Ser)) had an inconclusive result (<0.2 MVP score and clustering with reference controls), while another case (with variant c.3964G>C, p.(Gly1322Arg)) was classified as negative for IDDAM (MVP score of 0 and clustering with reference controls). However, the MDS plot showed a mirror image pattern of IDDAM signature samples—suggesting a possible gain of function allele instead of the usual haploinsufficiency. For the phenotype of these particular individuals, see the section “Phenotype of individuals with a VUS”. See Supplemental Table 2 for all EpiSign results.

### Phenotype of individuals with a (likely) pathogenic variant in *CHD8*

Based on the clinical features and their frequencies, Table 1 was designed to give a clear overview of the most frequently reported phenotypic abnormalities. All clinical data, including individual patient information, are shown in Supplementary Table 1. In this section, we will describe the phenotype of the 101/106 individuals with a (likely) pathogenic variant in *CHD8*—individuals with a VUS are excluded from this descriptive analysis.

**Table 1:** Overview of phenotypic abnormalities described in individuals with a (likely) pathogenic variant in *CHD8*.

Article	Bernier et al. [4]	Alotaibi et al. [23]	An et al. [9]	Douzgou et al. [6]	Han et al. [24]	Lee et al. [25]	Merner et al. [26]	Kimura et al. [27]	Stolerman et al. [28]	Tran et al. [29]	Wang et al. [30]	Ostrowski et al. [7]	O’Roak et al. [31]	D’Gama et al. [32]	Cappi et al. [33]	Talkowski et al. [34]	Wang et al. [35]	New cohort	Total
Sex (male/female)	11/4	1/0	3/1	8/2	1/0	0/2	1/0	0/1	1/0	1/0	2/0	16/3	6/1	1/0	1/0	0/1	1/1	20/11	74/27
Head circumference (at birth) > P98	0/1	0/1	3/4	U	U	2/2	U	U	U	U	0/2	0/1	U	U	U	U	U	3/4	8/15 (53%)
Head circumference > P98	10/15	0/1	0/4	5/10	U	2/2	0/1	U	0/1	U	1/2	11/18	1/4	U	U	1/1	2/2	13/27	46/88 (52%)
Height (at birth > P98	U	0/1	0/4	U	U	U	U	U	0/1	U	0/2	0/1	U	U	U	U	U	0/7	0/16 (0%)
Height > P98	6/14	0/1	0/4	6/10	0/1	1/2	0/1	U	U	U	2/2	15/19	2/5	U	U	U	2/2	5/17	39/78 (50%)
Weight (at birth) > P98	U	0/1	0/4	0/7	U	U	U	U	0/1	0/1	0/2	1/5	U	U	U	U	U	5/17	6/38 (16%)
Weight > P98	3/13	0/1	0/4	2/8	1/1	U	0/1	U	U	U	1/2	11/18	0/5	U	U	U	2/2	4/16	24/71 (34%)
Motor delay (HP:0001270)	0/15	U	0/3	0/10	U	2/2	0/1	U	0/1	U	0/2	U	U	U	U	U	0/2	14/17	16/53 (30%)
Speech delay (HP:0000750)	1/14	0/1	0/4	0/9	0/1	0/1	0/1	0/1	0/1	0/1	0/2	U	0/7	U	U	U	0/2	10/16	11/61 (18%)
Intellectual disability (HP:0001249)	9/15	U	3/3	7/10	1/1	0/2	1/1	1/1	0/1	U	U	18/18	0/6	0/1	0/1	U	2/2	13/19	55/81 (68%)
Abnormality of prenatal development or birth (HP:0001197)	U	U	U	U	U	U	U	U	U	U	U	1/1	U	U	U	U	1/1	4/4	6/6 (100%)
Neurological abnormality (HP:0000707)	13/13	U	2/2	9/10	U	2/2	1/1	U	U	U	1/1	19/19	U	1/1	U	U	2/2	23/24	73/75 (97%)
Seizures (HP:0001250)	3/13	U	1/2	4/10	U	0/2	0/1	U	U	U	0/1	2/19	U	1/1	U	U	0/2	2/24	13/75 (17%)
Neurodevelopmental delay (HP:0012758)	1/13	U	0/2	0/10	U	2/2	0/1	U	U	U	0/1	0/19	U	0/1	U	U	0/2	19/24	22/75 (29%)
Hypotonia (HP:0001252)	2/13	U	0/2	2/10	U	2/2	1/1	U	U	U	0/1	7/19	U	0/1	U	U	1/2	7/24	22/75 (29%)
Morphological central nervous system abnormality (HP:0002011)	2/13	U	0/2	4/10	U	0/2	0/1	U	U	U	1/1	1/19	U	0/1	U	U	0/2	3/24	11/75 (15%)
Neurological speech impairment (HP:0002167)	3/13	U	0/2	0/10	U	0/2	0/1	U	U	U	0/1	0/19	U	0/1	U	U	0/2	9/24	12/75 (16%)
Involuntary movements (HP:0004305)	4/13	U	2/2	1/10	U	0/2	1/1	U	U	U	1/1	0/19	U	0/1	U	U	2/2	2/24	13/75 (17%)
Abnormality of the brain (HP:0012443)	0/13	0/1	0/4	4/10	U	0/2	U	U	U	0/1	2/2	1/19	U	U	U	0/1	0/2	3/11	10/66 (15%)
Behavioural problems (HP:0000708)	15/15	1/1	4/4	9/10	1/1	0/2	1/1	1/1	1/1	1/1	2/2	13/19	7/7	1/1	1/1	1/1	2/2	23/25	84/95 (88%)
Stereotypy (HP:0000733)	5/15	1/1	4/4	1/10	0/1	0/2	1/1	0/1	1/1	0/1	2/2	0/19	0/7	0/1	0/1	0/1	2/2	3/24	20/94 (21%)
Abnormal aggressive, impulsive or violent behaviour (HP:0006919)	1/15	1/1	3/4	2/10	0/1	0/2	0/1	0/1	1/1	0/1	0/2	3/19	0/7	0/1	0/1	0/1	0/2	5/24	16/94 (17%)
Insomnia (HP:0100785)	7/15	0/1	4/4	3/10	0/1	0/2	0/1	1/1	0/1	0/1	0/2	0/19	0/7	0/1	0/1	0/1	0/2	4/25	19/95 (20%)
Sleep disturbance (HP:0002360)	10/15	0/1	4/4	4/10	0/1	0/2	1/1	1/1	0/1	0/1	0/2	0/19	0/7	0/1	0/1	0/1	0/2	7/24	27/94 (29%)
Short attention span (HP:0000736)	11/15	1/1	1/4	4/10	1/1	0/2	1/1	0/1	1/1	1/1	1/2	3/19	0/7	0/1	0/1	0/1	2/2	3/24	30/94 (32%)
Poor eye contact (HP:0000817)	4/15	1/1	4/4	0/10	0/1	0/2	0/1	0/1	1/1	1/1	2/2	0/19	0/7	0/1	0/1	0/1	0/2	2/24	15/94 (16%)
Autism spectrum disorder or autistic behaviour (HP:0000729)	13/15	1/1	4/4	8/10	0/1	0/2	1/1	1/1	1/1	1/1	2/2	9/19	7/7	1/1	0/1	1/1	2/2	19/24	71/94 (76%)
Abnormal social behaviour (HP:0012433)	12/15	1/1	4/4	3/10	0/1	0/2	0/1	0/1	1/1	1/1	2/2	1/19	0/7	0/1	0/1	0/1	0/2	4/24	29/94 (31%)
Impaired social interactions (HP:0000735)	12/15	1/1	4/4	2/10	0/1	0/2	0/1	0/1	1/1	1/1	2/2	1/19	0/7	0/1	0/1	0/1	0/2	2/25	26/95 (27%)
Impairment in personality functioning (HP:0031466)	4/15	0/1	3/4	2/10	0/1	0/2	1/1	0/1	0/1	0/1	2/2	0/19	0/7	0/1	0/1	0/1	1/2	3/24	16/94 (17%)
Repetitive compulsive behaviour (HP:0008762)	1/15	1/1	4/4	1/10	0/1	0/2	1/1	0/1	1/1	0/1	2/2	0/19	0/7	0/1	0/1	0/1	2/2	0/24	13/94 (14%)
Abnormality of the forehead (HP:0000290)	3/3	U	3/3	U	U	U	U	U	U	U	U	U	U	U	U	U	U	11/12	17/18 (94%)
Abnormality of the eye (HP:0000478)	8/15	0/1	4/4	1/1	U	2/2	1/1	U	U	U	0/1	2/19	U	U	U	1/1	U	11/18	30/63 (48%)
Abnormality of the ocular adnexa (HP:0032039)	7/15	1/1	0/4	0/1	U	2/2	0/1	U	U	U	0/1	0/19	U	U	U	0/1	U	8/18	18/63 (29%)
Hypertelorism (HP:0000316)	6/15	0/1	4/4	0/1	U	2/2	0/1	U	U	U	0/1	0/19	U	U	U	0/1	U	5/18	17/63 (27%)
Abnormality of the mouth (HP:0000153)	2/11	1/1	1/4	U	U	U	1/1	U	U	U	0/1	1/19	U	U	U	0/1	U	9/18	15/56 (27%)
Abnormal oral morphology (HP:0031816)	2/11	1/1	1/4	U	U	U	1/1	U	U	U	0/1	1/19	U	U	U	0/1	U	8/18	14/56 (25%)
Abnormality of the nose (HP:0000366)	2/15	1/1	4/4	U	U	U	1/1	U	U	U	0/1	0/19	U	U	U	0/1	U	10/18	18/60 (30%)
Abnormality of the ear (HP:0000598)	5/15	0/1	4/4	U	U	U	1/1	U	1/1	U	0/1	1/19	U	U	U	1/1	U	6/18	19/61 (31%)
Abnormality of the dentition (HP:0000164)	U	U	1/1	1/1	U	2/2	U	U	1/1	U	U	1/1	U	U	U	U	U	4/5	10/11 (91%)
Musculoskeletal abnormality (HP:0033127)	12/15	U	1/1	4/4	U	2/2	U	U	0/1	U	0/1	16/19	U	U	U	1/1	U	13/18	49/62 (79%)
Abnormality of limbs (HP:0040064)	5/15	U	0/1	1/4	U	2/2	U	U	0/1	U	0/1	3/19	U	U	U	0/1	U	9/18	20/62 (32%)
Abnormality of the supraorbital ridges (HP:0100538)	6/15	U	1/1	0/4	U	0/2	U	U	0/1	U	0/1	0/19	U	U	U	1/1	U	2/18	10/62 (16%)
Abnormality of the musculature (HP:0003011)	2/15	U	0/1	0/4	U	2/2	U	U	0/1	U	0/1	7/19	U	U	U	0/1	U	3/18	14/62 (23%)
Abnormal foot morphology (HP:0001760)	4/15	U	0/1	1/4	U	2/2	U	U	0/1	U	0/1	2/19	U	U	U	0/1	U	8/18	17/62 (27%)
Abnormality of the curvature of the vertebral column (HP:0010674)	1/15	U	0/1	1/4	U	2/2	U	U	0/1	U	0/1	2/19	U	U	U	0/1	U	3/18	9/62 (15%)
Abnormality of the hand (HP:0001155)	2/15	U	0/1	0/4	U	0/2	U	U	0/1	U	0/1	3/19	U	U	U	0/1	U	5/18	10/62 (16%)
Abnormal joint morphology (HP:0001367)	1/15	U	0/1	0/4	U	2/2	U	U	0/1	U	0/1	1/19	U	U	U	0/1	U	3/18	7/62 (11%)
Pes planus (HP:0001763)	2/15	U	0/1	1/4	U	2/2	U	U	0/1	U	0/1	2/19	U	U	U	0/1	U	4/18	11/62 (18%)
Abnormality of the digestive system (HP:0025031)	12/15	U	4/4	2/2	U	2/2	U	1/1	1/1	U	2/2	1/19	U	U	U	U	2/2	8/18	35/66 (53%)
Diarrhoea (HP:0002014)	4/15	U	0/4	0/2	U	2/2	U	1/1	1/1	U	1/2	0/19	U	U	U	U	0/2	1/18	10/66 (15%)
Constipation (HP:0002019)	9/15	U	3/4	1/2	U	2/2	U	0/1	0/1	U	2/2	0/19	U	U	U	U	0/2	5/18	22/66 (33%)
Cardiac abnormality (HP:0001627)	0/14	U	U	U	U	2/2	U	U	U	U	U	0/19	U	U	U	0/1	U	1/15	3/51 (6%)
Abnormality of the respiratory system (HP:0002086)	2/2	U	U	U	U	2/2	U	U	U	U	U	U	U	U	U	U	U	3/6	7/10 (70%)
Abnormality of the genitourinary system (HP:0000119)	2/14	U	2/2	1/1	U	U	U	U	U	U	1/1	1/19	U	U	U	U	U	3/18	10/55 (18%)
Abnormality of the genital system (HP:0000078)	1/14	U	2/2	1/1	U	U	U	U	U	U	1/1	1/19	U	U	U	U	U	3/18	9/55 (16%)
Abnormal reproductive system morphology (HP:0012243)	1/14	U	2/2	1/1	U	U	U	U	U	U	1/1	1/19	U	U	U	U	U	3/18	9/55 (16%)
Abnormality of the integument (HP:0001574)	2/14	U	U	U	U	U	U	U	U	U	0/1	1/19	U	U	U	U	U	8/18	11/52 (21%)
Visual impairment (HP:0000505)	0/14	0/1	U	0/1	U	0/2	0/1	U	U	U	U	0/19	U	U	U	U	U	0/18	0/56 (0%)
Abnormal hearing (HP:0000364)	1/14	0/1	U	1/1	U	U	0/1	U	U	0/1	0/2	0/19	U	U	U	U	U	1/18	3/57 (5%)
Abnormality of the immune system (HP:0002715)	1/14	U	U	0/1	U	U	U	U	U	U	U	0/19	U	U	U	U	U	3/17	4/51 (8%)
Abnormality of the endocrine system (HP:0000818)	0/14	U	U	0/1	U	U	U	U	U	U	U	1/19	U	U	U	U	U	4/17	5/51 (10%)
Abnormality of the metabolic system (HP:0001939)	3/14	U	U	1/1	U	2/2	U	U	U	U	U	0/19	U	U	U	U	U	3/17	9/53 (17%)
Hyperbilirubinemia (HP:0002904)	1/14	U	U	1/1	U	2/2	U	U	U	U	U	0/19	U	U	U	U	U	2/17	6/53 (11%)
Neoplasia (HP:0002664)	1/14	U	U	3/3	U	U	U	U	U	U	U	1/19	U	U	U	U	U	1/18	6/54 (11%)

*U* unknown.

Variants in *CHD8* cause a broad spectrum of symptoms (Table 1). The main clinical features of IDDAM are intellectual disability with an overgrowth phenotype in combination with behavioural abnormalities. To be exact, the vast majority of the individuals (68%, 55/81) had an intellectual disability, varying from mild (48%), moderate (24%) and severe in (28%) individuals who had severity specified. Regarding overgrowth, macrocephaly is a striking feature that was common, at birth (53%, 8/15) and at the age of examination (52%, 46/88), as well as tall stature (50%, 39/78) and being overweight (34%, 24/71). Behavioural abnormalities (including autism spectrum disorder) were present in 88% of individuals (see Table 2 and section “Behavioural phenotype ” below as well).

**Table 2:** Behavioural phenotype in individuals with a (likely) pathogenic variant in *CHD8*.

	Positive	Total evaluated	Percentage
Behavioural problems (HP:0000708)	84	95	88
Autism spectrum disorder or autistic behaviour (HP:0000729)	71	94	76
Short attention span (HP:0000736)	30	94	32
Abnormal social behaviour (HP:0012433)	29	94	31
Sleep disturbance (HP:0002360)	27	94	29
Impaired social interactions (HP:0000735)	26	95	27
Stereotypy (HP:0000733)	20	94	21
Insomnia (HP:0100785)	19	95	20
Abnormal aggressive, impulsive or violent behaviour (HP:0006919)	16	94	17
Impairment in personality functioning (HP:0031466)	16	94	17
Poor eye contact (HP:0000817)	15	94	16
Repetitive compulsive behaviour (HP:0008762)	13	94	14
Irritability (HP:0000737)	5	94	5
Hyperactivity (HP:0000752)	4	94	4
Psychosis/Schizophrenia (HP:0000709/HP:0100753)	3	94	3
Excessive daytime somnolence (HP:0001262)	1	94	1
Inflexible adherence to routines or rituals (HP:0000732)	1	94	1
Obsessive-compulsive behaviour (HP:0000722)	1	94	1

Looking at other clinical features that stood out, gastrointestinal problems were common. Constipation (33%, 22/66) and diarrhoea (15%, 10/66) are specifically reported recurrent abnormalities. A minor cardiac abnormality was reported three times (6%, 3/51): a heart murmur, mitral regurgitation, and a patent foramen ovale, all at once. An abnormality of the respiratory system was described in seven individuals (70%, 7/10), mainly respiratory distress (in three individuals). Immunological problems (in 8%, 4/51) included gluten intolerance in two individuals, recurrent otitis media in one, and neutropenia in one. Looking at the endocrine system, abnormalities were described in five individuals (10%, 5/51)—mainly precocious puberty and hypothyroidism, both in two individuals. Finally, in nine individuals, abnormalities of the metabolic system were found (17%, 9/53). Specifically, hyperbilirubinemia was described in six of 53 individuals (11%). Neoplasia was seen in six individuals (11%, 6/54): glabellar hemangioma in three, fibroma in one, fibrosarcoma in one, and uterine leiomyoma in one individual.

#### Behavioural phenotype

A wide spectrum of behavioural abnormalities was reported, with the most noted behavioural abnormality being autism spectrum disorder or autistic behaviour (76%, 71/94, see Table 2 for all reported behavioural abnormalities). Furthermore, other psychiatric diagnoses such as psychosis/schizophrenia (in three individuals, 3%) and repetitive compulsive behaviour (in 13 individuals, 14%) were relatively common. All in all, a large majority (88%) of the individuals included in this study had some form of behavioural problems.

### Phenotype of individuals with a VUS

For five individuals, consisting of one family with three affected individuals and two sporadic cases, a variant of unknown significance (VUS) in *CHD8* was reported (Table 1).

The family consisted of two affected sibs (brother and sister) and their father, in whom a *CHD8* (c.1772T>A, p.(IIe591Lys)) VUS was reported. All were overweight and had tall stature. Both sibs were macrocephalic (head circumference for father unknown); however, no developmental or behavioural problems were reported except for some anxiety in the brother. Some dysmorphic features were seen, mainly a long face, tubular nose, and arachnodactyly. In addition, hypermobility and skin striae were noted. These clinical features seem to fit the *CHD8* phenotype well, since being overweight (present in 34% of individuals with a likely pathogenic variant in *CHD8*), tall stature (50%), macrocephaly (52%) and musculoskeletal abnormalities (79%) are all common symptoms. However, in this case, behavioural problems are not present—while these are common in individuals (88%) with a pathogenic variant in *CHD8*.

For two others, pathogenicity was questionable after EpiSign analyses (see section “Phenotype of individuals with a VUS”). Phenotypic information for these individuals was unfortunately scarce: one individual (c.3964G>C, p.(Gly1322Arg)) had ID, global development delay, behavioural problems, and scoliosis. The other individual with variant c.1928T>G, p.(Ile643Ser) was noted to have developmental delay, hypotonia, a sensory condition, and unspecified dysmorphic features.

### Genotype–phenotype correlation and disease severity

A Mann–Witney *U* test was conducted to compare the severity of the phenotypes of males and females and of individuals with missense variant and individuals with another variant. No statistically significant differences were observed between males and females (*p* = 0.93). However, individuals with a missense variant were less severely affected than individuals with other variants (median De Vries score 1.0 vs. 3.0; *p* = 0.046).

## Discussion

### Summary and relevance

This study provides an overview of all phenotypic abnormalities present in 106 individuals with IDDAM caused by a variant in *CHD8*. By collecting data from 36 novel individuals and aggregating these data with information on 70 individuals published in 17 articles, we provide insight into the broad spectrum of phenotypic abnormalities caused by a (likely) pathogenic variant in *CHD8*. Therefore, this study can provide recommendations for physical examination and surveillance.

Furthermore, we confirmed the pathogenicity of missense and frameshift/nonsense variants by comparing profiles to established methylation signatures, and improve the current EpiSign technology by significantly increasing the number of individuals in the dataset. This will make it easier to reclassify VUSs in *CHD8* in the future.

Consistent with previous studies and the function of *CHD8*, we found pathogenic and likely pathogenic variants in *CHD8* cause a broad spectrum of phenotypic abnormalities [4, 6, 7]. In these studies, ASD, overgrowth, constipation, developmental delay, and intellectual disability were already linked to disease-associated variants in *CHD8*. Douzgou et al. [6] added, among other features, hypotonia and seizures to this list, and Ostrowski et al. [7] mentioned skeletal abnormalities. In this study, the above symptoms and more phenotypic abnormalities were linked to IDDAM. Genitourinary abnormalities, now observed in 18% of the individuals, had previously not been described as a commonly found feature in individuals with a pathogenic or likely pathogenic variant in *CHD8* before. Next to the broad phenotypic spectrum, we found large variability between individuals.

### Recommendations

By looking at the frequencies in which specific abnormalities were observed, advice can be given regarding examination and follow-up in individuals with IDDAM (Table 3). Since developmental delay and ID are frequently described, we recommend developmental evaluation at an early stage so referral for speech and/or physical therapy can be made, if necessary. We also recommend neurological screening with special attention for hypotonia, seizures, and brain imaging for abnormalities (mainly ventriculomegaly and white matter abnormalities). In most individuals, behavioural abnormalities were noted. Therefore, we recommend screening individuals with IDDAM for behavioural abnormalities such as ASD, short attention span, psychiatric conditions, abnormal fear/anxiety-related behaviour and emotion/affective behaviour, problems with sleeping, abnormal social behaviour, repetitive, compulsive behaviour, and other striking behavioural characteristics. As recommended by the American Academy of Paediatrics and the Center for Disease Control and Prevention, ASD screening is recommended in children at 18 and 24 months of age because these are critical times for early social and language development, thus providing a window of opportunity for more effective early intervention for ASD [20]. The same applies to other behavioural abnormalities. A diagnosis can lead to improved parent education, and therefore, behaviours can be better understood and guided.

**Table 3:** Recommendations regarding follow-up and examination of individuals with a variant in *CHD8*.

System	Evaluation/concern	Clarification
Growth	Assessment of growth parameters	Tall stature and macrocephaly are reported frequently.
Neurological	Developmental evaluation	Screening for motor and speech delay and general cognitive abilities/intellectual disability.
	Neuropsychiatric evaluation	There is a higher risk for ASD, short attention span, psychiatric conditions, abnormal fear/anxiety-related behaviour and emotion/affective behaviour, sleeping problems, abnormal social behaviour and repetitive compulsive behaviour. Preferably performed at a young age.
	Neurological evaluation	In this evaluation, there should be special attention to hypotonia.
	Magnetic resonance imaging (MRI) of the brain	Consider screening for structural abnormalities of the brain if indicated neurological features.
	Electroencephalography (EEG)	Advised on indication when there is suspicion on seizures.
Gastrointestinal		Special attention for constipation.
Musculoskeletal		Clinical examination for abnormality of the curvature of the vertebral column, jaw abnormalities and pes planus. X-ray when indicated after the examination.
Genitourinary	Ultrasound for abnormalities of the kidneys and genital system.	Should be performed at least once at diagnosis with follow-up if abnormalities are present
Other	Screening for neonatal icterus	Higher risk for neonatal hyperbilirubinemia: Explanation/awareness for parents.
	Glabellar skin	Alertness for glabellar haemangioma
	Consultation of clinical geneticist/genetic counsellor	

There is a higher risk for constipation, and adjustments in diet or medication can be considered based on the burden of constipation. Skeletal abnormalities should be kept in mind and regularly screened for (mainly abnormality of the curvature of the vertebral column, jaw abnormalities, and pes planus) using regular clinical examinations and x-rays when needed. Furthermore, we recommend screening for anomalies of dentition. Also, growth should be monitored, in comparison with measurements of parents, and stagnated prematurely in puberty when desirable. We also recommend a single renal ultrasound for abnormalities of the genitourinary system. If the diagnosis has been established prenatally, there should be extra attention to neonatal hyperbilirubinemia. Although neonatal hyperbilirubinemia was not observed in most of the individuals, it was more prevalent than in the overall population, so parents should be informed about recognizing icteric symptoms, and healthcare professionals can pay extra attention to these symptoms in the first weeks after birth. Glabellar haemangioma was reported in three individuals, and therefore a clinician should be extra alert for this possible anomaly during physical examination.

### Methylation signature and VUSs

For 11 of the 13 patients for whom the material was available, we have established a positive IDDAM methylation signature according to the EpiSign analysis. This confirms that both missense and frameshift/nonsense variants can be pathogenic. The inclusion of these new data to the EKD will expand the current reference cohort and aid in the refinement of the episignatures, leading to a more effective classification of VUSs. Furthermore, no difference was observed in signature between missense and frameshift/nonsense variants, further confirming that haploinsufficiency is indeed the mechanism that leads to the phenotype of IDDAM. Interestingly, the methylation profile of one patient (with variant c.3964G>C, p.(Gly1322Arg)) suggested a mirror image, seen in signatures of deletions vs. duplications in other syndromes. This suggests a possible gain of function allele—not previously described in IDDAM. Phenotypically, this patient presents with intellectual disability, global development delay, behavioural problems, and scoliosis. Unfortunately, growth parameters were not available for this particular individual. While the combination of behavioural issues with scoliosis is quite striking and corresponds with the phenotype of the individuals with pathogenic and likely pathogenic variants in *CHD8*, the phenotype is not specific enough to warrant a definitive conclusion on the variant’s pathogenicity. However, one might conclude that the phenotype of this individual is not a mirror image of the general IDDAM phenotype. Another indication that haploinsufficiency might not be the only mechanism in play in IDDAM, is that individuals with a missense variant have a significantly lower De Vries score than individuals with a variant in *CHD8* of another type (1.0 vs. 3.0 on median; *p* = 0.046). Possibly, a difference in the level of loss-of-function in missense variants leads to a less severe phenotype. To confirm (or disprove) possible other alternative pathophysiological mechanisms than haploinsufficiency, further functional tests are needed. A possible approach could be to look at downstream functional effects of *CHD8:* others have previously shown that *CHD8* inhibits the Wnt–β-catenin signalling pathway (by promoting the combining of β-catenin and histone H)—with a higher associated Wnt response in a dominant negative *CHD8* mutant [2]. One could design experiments similar to those by Nishiyama et al. and investigate the associated Wnt response: a gain-of-function effect of a variant in *CHD8* should lead to further downregulation of the Wnt–β-catenin signalling pathway.

Looking at the phenotype of the individual with an inconclusive methylation signature (c.1928T>G, p.(Ile643Ser)), developmental delay, hypotonia, a sensory condition, and dysmorphic features were reported. Interestingly, no behavioural problems or skeletal abnormalities were seen, supporting the conclusion that this variant might be benign rather than pathogenic. Still, the IDDAM phenotype can be mild, so pathogenicity cannot be ruled out.

Finally, we included one family in this study, with the variant reported as a VUS as well. These individuals exhibit overgrowth—and musculoskeletal symptoms corresponding with the IDDAM features. However, no ASD or similar behavioural problems were noted—lowering the suspicion of pathogenicity of this specific variant. Overall, the phenotype seems to correspond with that of IDDAM, and methylation analysis confirms the pathogenicity of this particular variant. This is an example of the power of EpiSign: when combined with phenotypic comparison, variants of unknown significance can be reclassified, leading to more diagnoses. Of note, since this variant was only determined as pathogenic after phenotypic comparison and EpiSign analysis, the phenotype of this family is not included in the main analysis of (likely) pathogenic variants in this study.

### Limitations of this study

In this study, all individuals with a (likely) pathogenetic variant in *CHD8* described in the literature were included to create a complete overview of all currently available information. Symptoms such as ASD, DD, and ID are frequently reported. It needs to be taken into account that several studies included in this review genetically analysed a cohort of individuals with ASD, DD, or ID. This might have caused selection bias, and the number of individuals with ASD, DD, and ID might not be representative of the whole group of *CHD8* individuals.

This review includes 76 males and 30 females and bias towards males has been acknowledged in a previous study [7]. An explanation might be that females with a variant in *CHD8* are less severely affected and therefore less frequently diagnosed, less frequently genetically tested, and reported in the literature. This hypothesis is supported by mice experiments suggesting a sex-specific effect on transcriptional regulation and phenotype, with male mice being more affected [21]. However, we could not find a statistically significant difference in phenotype severity between males and females, questioning whether this difference in phenotype between the sexes is present in humans as well. It was previously noted that variants in *CHD8* in the literature are often detected by genetically analysing a patient cohort with a symptom frequently present among individuals with a pathogenic variant in *CHD8*, such as ASD. If a variant in *CHD8* causes less ASD in females, they are not included in these cohorts in the first place, which causes inclusion and detection bias. Particularly, since ASD is known to be more common in men and presents differently in women, it may therefore be less frequently diagnosed [22]. In future studies, it would be interesting to look at *CHD8* individuals independently from pre-screened symptoms to include individuals with milder symptoms and compare males and females again in a larger cohort. A difficulty might be a low rate of variants in *CHD8* found in a non-pre-screened cohort. A limitation of using data from previously published individuals is that only mentioned symptoms were collected on the HDG website. Therefore, for some phenotypic abnormalities, it is not entirely certain if they were absent or only not mentioned. In clinical practice, symptoms might be present or absent in more convincing numbers than presented in this study.

## Conclusion

In this review, we present the phenotypic data of 106 individuals with a variant in *CHD8*. We show that IDDAM consists of a wide range of symptoms in multiple organ systems, most frequently involving overgrowth, developmental delay, neurological, behavioural/psychiatric, gastrointestinal, musculoskeletal, and genitourinary abnormalities. Considerable variation between individuals was seen. Although more males than females were included, no significant difference in phenotype between the sexes was observed. However, individuals with a missense variant were less severely affected than individuals with a variant of another type. Recommendations for follow-up of individuals with IDDAM were provided, which will be of significant value to clinicians as well as to the individuals and their families.

## Supplementary information

Clinical details of all included individuals

Methylation analysis

## Data Availability

Genetic counsellors, other clinicians, individuals, and their families will have access to the data described in this paper directly online on the Human Disease Genes (HDG) website series (www.humandiseasegenes.info/CHD8) [13]. It will be available in a clear graphical summary. Since it is likely that we still do not know everything about the phenotype caused by a variant in *CHD8*, it is essential that we continue to collect data. Through the HDG website series, data can be added continuously (www.humandiseasegenes.nl/chd8/professionals/upload-clinical-information), resulting in an up-to-date dataset.
